# Searching for the Metaverse: Neuroscience of Physical and Digital Communities

**DOI:** 10.1089/cyber.2023.0040

**Published:** 2024-01-08

**Authors:** Giuseppe Riva, Brenda K. Wiederhold, Fabrizia Mantovani

**Affiliations:** ^1^ Applied Technology for Neuro-Psychology Lab, IRCCS Istituto Auxologico Italiano, Milan, Italy.; ^2^ Humane Technology Lab, Università Cattolica del Sacro Cuore, Milan, Italy.; ^3^ Virtual Reality Medical Center, La Jolla, California, USA.; ^4^ Virtual Reality Medical Institute, Brussels, Belgium.; ^5^ Centre for Studies in Communication Sciences “Luigi Anolli” (CESCOM), Department of Human Sciences for Education “Riccardo Massa,” University of Milano Bicocca, Milan, Italy.

**Keywords:** metaverse, neuroscience, community, digital communities, presence, co-presence, social capital, we-mode

## Abstract

What distinguishes real-world communities from their online counterparts? Social and cognitive neuroscience research on social networks and collective intentionality will be used in the article to answer this question. Physical communities are born in places. And places engage “we-mode” neurobiological and cognitive processes as behavioral synchrony, shared attention, deliberate attunement, interbrain synchronization, and so on, which create coherent social networks of very different individuals who are supported by a “wisdom of crowd.” Digital technologies remove physical boundaries, giving people more freedom to choose their activities and groups. At the same time, however, the lack of physical co-presence of community members significantly reduces their possibility of activating “we-mode” cognitive processes and social motivation. Because of this, unlike physical communities that allow interaction between people from varied origins and stories, digital communities are always made up of people who have the same interests and knowledge (communities of practice). This new situation disrupts the “wisdom of crowd,” making the community more radical and less accurate (polarization effect), allowing influential users to wield disproportionate influence over the group's beliefs, and producing inequalities in the distribution of social capital. However, a new emergent technology—the Metaverse—has the potential to reverse this trend. Several studies have revealed that virtual and augmented reality—the major technologies underlying the Metaverse—can engage the same neurobiological and cognitive “we-mode” processes as real-world environments. If the many flaws in this technology are fixed, it might encourage people to engage in more meaningful and constructive interactions in online communities.

“We are each of us angels with only one wing, and we can only fly by embracing one another.”

Luciano De Crescenzo

## Introduction

Humans are social beings. And it is true that we interact with other people throughout most of our lives. We deal with them directly or collaboratively. While some people we grow to love, others we only engage in casual eye contact with as we cross the street. However, despite this wide range of interactions and actions, we may categorize the Others into two major groups, which are represented by the two plural pronouns: the first person (We/Us) and the third person (They/Them).

Where is the distinction? To put it simply, we might say that, although in “They,” the Other/s (Them) exist in opposition to the I, in “We,” there is both the I and the Other/s (Us). Three essential concepts are not covered by this definition, although. How does our interaction with others differ depending on whether it is Us or They? Then, how do other people become either Us or Them? Why do we have a Us and a Them, then?

We use the term “social network” to refer to the group of people with whom an individual has some sort of social connection, whether it be familial, platonic, or professional ties. However, social networks are more than just a collection of people in a given environment that influences our actions.

On one side, we internalize the experience made in them, incorporating it in our sense of self. In simpler words, our reflexive activity about the experience we do in our social networks defines our “social identity”^1^: the “knowledge that [we] belong to certain social groups together with some emotional and value significance to [us] of this group membership” (p. 31).

On the other side, social networks enrich our lives in different ways.^2,3^ First, they improve the quality of our personal experience by providing social companionship and emotional bonding (individual level). Second, they allow us to achieve competences and goals that would otherwise be unattainable through collaborative learning and intellectual stimulation (behavioral level). Third, they organize and enhance our agency by providing trust and norms of collective action (institutional level). Fourth, the above processes generate a sense of community that is the result of the shared history of its members, and it is where the sense of social identity develops (community level).

Social sciences define these advantages as “social capital”^4,5^: the benefits accruing to individuals by virtue of their long-term ties with others (see Table 1 for further details).

**Table 1.** The Different Components of Social Capital

According to the specific benefits generated, three types of social capital can be distinguished^81^: bonding, bridging, and linking social capitals. Strong links between members of a community who view themselves as similar develop bonding social capital. The fundamental result of this social capital is the sensation of social cohesion: a strong feeling of friendship, love, or shared ideas and experiences that bind members of a community together. Bridging social capital is instead formed by bonds of respect and mutuality between people of a community who are not sociodemographically similar. The primary effect of this social capital is increased information diffusion within and between groups, providing access to a broader range of skills and opportunities that would not otherwise be available. Finally, bonds of respect and mutuality between members of a community with unequal authority develop linking social capital. The main result of this social capital is enhanced access to financial and political resources. A successful society requires all three types of social capital and achieving the correct balance between them is crucial for its survival and growth.

Moreover, social networks are not all created equal. Communities are stable social networks based on physical closeness or shared interests, whereas groups are more nimble social networks based on shared, but short-lived passions or concerns.^6^ Specifically, if groups have specific goals that are circumstantial and defined in time, communities can extend beyond a precise period and sometimes even a precise place.

According to Putnam,^7^ communities are the main source of social capital. However, in the last decades, the meaning of this word has changed significantly. Before the introduction of communication technologies, communities were equated with neighborhoods—social networks, including people living near each other. However, the diffusion of the Internet and social media has generated digital communities that have only marginal links with physical places.

What are the differences between them? Unfortunately, so far, there is no easy answer to this question. Many scholars, from Wellman^8^ onward, argue that digital social networks have a greater reach than physical ones, allowing people to maintain more ties and establish more specialized interactions. On the other side, different researchers, from Dohény-Farina^9^ onward, contend that digital social networks are hastening the dissolution of community life by isolating us from real places.

In this article, we will attempt to answer these questions using recent research findings from social and cognitive neuroscience related to social networks and collective intentionality.

First, the article will argue that the difference between groups and communities, between “They/Them” and “We/Us,” is related to the different ways social actions are performed and experienced in them. Raimo Tuomela^10^ suggested that subjects in a social network act together in either the “I-mode” or the “we-mode.” When they behave in the “I-mode,” even if they act in a group, their commitment to the action is private and based on their personal aims. For example, if two people have the same destination, they can share a taxi instead of each getting their own.

What defines a community is instead the “we-mode”: working together as a “we” to advance the interests of “us.” In we-mode, individuals must intend to behave or have attitudes as a group for the same group purpose (collective intentions), and they must regard themselves as members of a community bonded by and committed to what is collectively recognized and subject to collective commitment in the group.

Furthermore, the article will use the recent research in neuroscience, both to explore the neurobiological underpinnings of the “we-mode” and to discuss the changes in the “we-mode” produced by the shift from physical to digital communities.

## Neuroscience of Physical Communities

The concept of physical community has always been linked to the concept of location in social psychology: physical communities are born in places. However, what is a place? According to the Merriam-Webster dictionary, a place is “a specific area,” “a particular part of a surface.” In other words, a defined space enclosed by boundaries.

However, boundaries do more than just define a place. They also provide the framework for building a physical community. First, boundaries identify a specific place that is different from other spaces and usually provide the ability to benefit from things—material objects, persons, institutions, and symbols—included in it.^11^ Second, boundaries constrain the action of individuals outside and inside the place. On one side, they act as borders by limiting access to people on the outside. On the other side, they act as cages by forcing the people inside to interact with each other even if they would not. In the next few pages, we explore the effects of these features on physical communities.

### Neuroscience of places

The importance of places and the boundaries that define them to the development of physical communities has recently been underscored by neuroscientific research (see Table 2 for further details).

**Table 2.** Neuroscience of Places

The discovery of the “place cells” in the hippocampus^82^ demonstrated that our brain contains different neurons activated when we occupy a specific location in our surroundings, remaining virtually silent elsewhere in the environment.^83^ The preferred location of the activation by a place cell is defined as the “place field” and reflects information concerning the distance and direction to environmental boundaries. Place cells, on the other hand, are not just for keeping track of one's own location. They are used to organize memories about specific locations^83^ and other individuals^84^ along dimensions like power and affiliation. Qasim et al.^85^ found that single neurons in the human entorhinal cortex change their spatial tuning to target relevant memories for retrieval. This result suggests that our brain encodes together both location information and episodic memories^86^ and that this process is crucial for the formation and consolidation of our autobiographical memory.^87^ Finally, an fMRI study^88^ used multivoxel pattern analysis to determine the neural areas in which events could be discriminated based on specific features. The results showed that events can be discriminated based on location, person, and object features. However, the location played a more consistent role in determining the neural representation of events^89^ providing a scaffold on which remembered and imagined events are constructed.^90^

fMRI, functional magnetic resonance imaging.

The synthesis of this literature suggests that the experience and development of self and social identity are both individually and collectively anchored in the relationship to places: we define who we are through the memory of the individual and social experiences that occur within the various places we attend.^12^ And these experiences encompass all the emotions, values, meanings, and symbols that we actively develop and adapt during our long-term relationship with a place.

The first experiential outcome of these processes is the feeling of “place attachment,”^13^ the bonding that occurs between individuals and their meaningful places. In fact, our autobiographical memory, by directly linking our experiences and their affective, cognitive, and behavioral components to a specific place, generates an enduring psychological bond that offers significant advantages to the individual^13^: not only a temporal or personal continuity but also survival, security, and goal support. Different studies on rats^14,15^ have shown that the neuropeptide oxytocin plays a significant role in the process by generating a reliable and robust preference for the environment with which it is repeatedly associated.

In physical communities, however, the bonding with a place is also a social process. When does this process turn from individual to social? According to neuroscience, this happens in two different steps. First, through collective behaviors, which generate an interbrain neural synchronization.^16^ Second, through the conversations about these collective behaviors, which build a collective memory within a shared space of meaning.^17^

### Neuroscience of social identity and collective actions

Collective behaviors, in general, revolve around the ability to^18,19^ (a) accept a shared frame of reference, (b) share knowledge relating to the object (s) or purpose (s) that is the behavior's aim, and lastly, (c) act jointly to achieve it.

However, as explained by the Empathy-Collective Action model,^19^ collective behavior requires either the perception of needs of others or an empathic concern toward them (or both). However, these requirements have an emotional cost for the individual: the aversive arousal elicited through emotional contagion^19,20^ that generates divergent affective reactions, especially distress (i.e., self-focused aversive feelings) and empathic concern (i.e., empathizing with those requiring support makes it difficult to disengage without seeking to relieve their distress). As suggested by Grossman,^21^ fearfulness is a key adaptive affective trait supporting human-unique levels of cooperative concern and care. In particular, it facilitates care-based responding and provisioning from, while concurrently increasing cooperation with relevant others.

In this process, a critical role is played by oxytocin. In particular, oxytocin regulates the salience of external cues^22^ by modulating approach/avoidance motivational tendencies and behaviors (see Table 3 for further details).^23^ Both physical touch^24^ and social vocalizations with relevant others^25^ release oxytocin in humans, improving their engagement in collective actions.

**Table 3.** The Social Role of Oxytocin

Oxytocin regulates the activity of mirror neurons,^91,92^ a group of sensorimotor neurons that fire not only when the individual performs an action but also when the individual passively observes another agent doing a comparable action. According to Ho et al.,^92^ oxytocin regulates a complex neurohormonal network involved in mentalization, attention and mirroring (oriented at perception–action integration), and emotional modulation^93^ that plays a critical role in bio-behavioral synchrony.^94^ When engaged by oxytocin, such a network has a socioattentive function that increases the relevance of the social context and the planning of collaborative activities.^92^ Furthermore, oxytocin appears to have a significant role in affecting the functioning of the anterior cingulate cortex and its projections to the nucleus accumbens, a circuit directly engaged in the development of empathetic reactions.^95,96^
To summarize, oxytocin regulates attention-orienting reactions to external contextual cues, boosting the motivational relevance of relevant persons and facilitating any cooperative conduct that involves them.^23^

However, what are the neurophysiological effects of collective action?

Different neuroscience studies explored the effects of interpersonal synchrony, the temporary alignment of periodic behaviors with another person.^26,27^ These studies underline that the main effect is the emergence of a brain “we-mode” through the synchronization of different neurophysiological parameters, including heart rate^27^ and neural oscillations^28^ that demonstrate an amplified awareness of the other participant. For example, Cacioppo et al.^26^ found that interpersonal synchrony also enhances the participant's ratings of perceived interpersonal synchrony and social affiliation with the partner. More recently, Shiraishi and Shimada^29^ replicated this result, suggesting that the sense of joint agency strongly reflects the interbrain synchronization, which depends on the quality of mutual cooperation during a joint action.

An important outcome of this process is the development of “we-representations,” which specify joint action outcomes at the group level,^30–32^ allowing the individual to predict, and eventually correct, the contribution of the partner's behavior to the shared goal achievement.^33,34^ Individuals engaging in a collaborative activity create a sense of agency^35^ through these representations, both individually (“I'm doing this”) and collectively (“We're doing this together”).

These effects, however, are not only limited to interpersonal synchrony but also happen during collective action. A growing body of studies using hyperscanning^36^—a new brain imaging technique that allows the simultaneous measurement of the activity of multiple brains—has revealed that collective behaviors influence the rhythmic patterns of neural activity (brainwaves) that enable the coordinated activity of the brain (see Table 4 for further details).^37^ In reality, synchronized neural oscillations physically coordinate people by controlling how and when their bodies move together.^38^

**Table 4.** Recent Hyperscanning Studies

Dikker et al.^97^ used EEG hyperscanning to evaluate the potential link between the collective behavior of a classroom—a typical physical community—and the interbrain synchrony between its members over 11 different school days. Their result shows that interbrain synchrony is a direct biomarker of the quality of interactions of the community. Specifically, it is directly connected to the social dynamics of the classroom: the higher the interbrain synchrony, the better the levels of engagement and social closeness. A similar result was reported by Reinero et al.,^28^ who used EEG hyperscanning to explore the effects of collective behaviors within small groups.
These studies also suggest that interbrain synchrony is the outcome of the “joint attention” of the community, the experience of a group of individuals who know that they are attending to something in common.^98^ In fact, joint attention tunes the neural oscillations to the temporal structure of the common context through eye contact and the exchange of glances (mutual gaze)^98^: “prior eye contact potentially creates a context for joint attention, which subsequently induces higher interbrain synchrony.” (p. R347).

EEG, electroencephalogram.

It is also important to note, that interbrain synchrony is also the basis for what Colombetti and Krueger define as an “extended affectivity,” the ability of emotions to extend beyond the individual's brain and body.^39^ Extended affectivity is what differentiates emotional contagion—feeling the same emotion as the other in a way that is neither highly self-involved nor other-directed in orientation—from empathy—feeling the same emotion in a way that is self-involved, but not other-directed in orientation—and from emotional sharing—feeling the same emotion in a way that is co-regulated and constitutively interdependent.

### Neuroscience of shared narratives and collective memories

Collective actions are only the first step toward a social community. In fact, individuals are progressively bound in social groups by sharing beliefs, emotions, memories, and norms between them.^17^ And this is achieved by shared narratives, expressive actions that “make present” the collective actions and their interpretation in a particular space and time to the members of the community.^40,41^ These narratives—that can be shared through texts, social interaction, performances, pictures, physical objects, and rituals—use a story to tell the community members important things about themselves. Specifically, they transform the autobiographical memories of collective actions into collective memories, bridging the individual with the community.

Recently, different human brain imaging studies explored how the sharing of biographical information and narrative stimuli produces an interbrain correlation at different levels (see Table 5 for further details).

**Table 5.** The Effects of Sharing Biographical Information and Narrations

First, as demonstrated by a recent fNIRS study^99^ during the mutual sharing of biographical information in a face-to-face setting, the spontaneous production and observation of facial displays (eye gaze and facial motion) generates a cross-brain synchrony in different brain areas. As suggested by Krueger,^43^ and Fanghella et al.,^100^ these cross-brain processes may generate a joint space—a “we-space” of action and meaning—that supports the interpersonal attunement between partners within the context of the common activity.
A second level of synchronizations is instead generated by the experience of an identical narrative stimulus. As demonstrated by Pérez et al.,^101^ when subjects are presented with the same auditory or audiovisual narrative, they experience a strong intersubject correlation of heart rate, facilitating the sharing of social emotions. Moreover, they generate the interbrain synchronization of brain activity. This synchronization has been observed using different neuroimaging modalities, including EEG,^102,103^ MEG,^104^ and functional near-infrared spectroscopy.^105^

MEG, magnetoencephalography.

Nevertheless, how are narratives represented in the brain? In the last decade, different Functional magnetic resonance imaging studies suggested that the development of narratives involves the same neural mechanisms underlying episodic memory formation.^42^ As explained by Milivojevic et al.,^42^ “We propose that this type of narrative-based contextual representation may serve to organize episodic memories into networks of related events, unrestricted by space or time, and may be the neural mechanism underlying autobiographical narrative construction.” (p. 12421).

In conclusion, social narratives allow an interactive form of space management^43^—the negotiation and management of “we-space”—encoding social beliefs as causal sequences connecting agents, places, and events. As explained by Krueger and Osler,^44^ “A we-space arises when individuals interact with one another in ways that create a felt sense of a shared space of possibilities. The notion of we-space emphasizes how certain interpersonal interactions are permeated by a sense of sharing a space with another. What marks a we-space is a sense of connectedness with the other.” (p. 218).

Through this process, generated by a “we-mode” of brain functioning, they link the individual's personal identity with the social one,^45^ allowing the process of social categorization (i.e., the perception of themselves and others in terms of particular social categories), social identification (i.e., the perception of sharing specific physical, social, and/or mental characteristics that define the individual as a member of a group), and social comparison (i.e., the perception of the relative value or social standing of a particular group and its members).

Moreover, social narratives also generate the “wisdom of crowd”: aggregated beliefs of large groups can be factually accurate even when individuals have inaccurate beliefs.^46^ In fact, (a) social narratives maximize the amount of information available for the belief; (b) reduce the potential impact of divergent sources or inaccurate information; and (c) increase the credibility and validity of the aggregation process by making it more ecologically representative.^47^

## Neuroscience of Digital Communities

Physical communities are constrained by the boundaries defined by space and time: we can interact with other members only if we are in the same place at the same time. Historically, to overcome these constrains, the members of physical communities used spatial mobility and various communicative tools, from letters to phones. More recently, information and communication technology have been actively used to support and meet the goals of a physical community. One of the most studied and successful examples of this approach is the Camfield Estates–MIT Creating Community Connections Project.^48,49^ Using three different models of community engagement with technology—a community network where state-of-the-art desktop technologies have been offered to every family, a community content delivered online, and a community technology center located on the premises in the community center—the project was able to improve the sense of community, to empower the members, thus increasing their self-sufficiency.

However, the general availability and ubiquity of digital communication are radically transforming the place-centered definition of community.

### From “Us” to “Them”: how communication technologies are undermining social experience

Communication technologies, by removing the physical boundaries that define a place, allow a greater freedom in the behavior of individuals. And this freedom has been further enhanced by the emergence of social media^50^: digital platforms that support information sharing, user-created content, and collaboration across people. As underlined by Nadkarni and Hofmann,^51^ social media are not used just as communicative tools. In fact, they answer two different needs: (a) the need to belong, affiliating with others and gaining social acceptance, and (b) the need for self-presentation through impression management.

In this view, communication in social media is significantly different from what happens in physical communities.^52^ By removing the physical limits that define a place, digital technologies allow for more freedom in individual behavior and community member selection. As demonstrated by different studies,^53,54^ this feature maximizes bridging social capital and facilitates the participation in social movements.

However, the lack of a physical space does not allow a direct link between autobiographical memory and the experience of social media. In this view, the meaning of social media experience is different depending on how it is used.^55^ When individuals use social media passively (consuming information), the effect of negative social comparison compromises memory, generating a lower social connection and higher stress.^56^ On the other side, the active use of social media (recording and sharing personal experiences) facilitates rehearsal and meaning-making, also improving memory retention.^57^

Furthermore, social media do not embody the co-presence of another agent, significantly disrupting the many we-mode processes we described in the previous section: behavioral synchrony, joint attention, intentional attunement, and so on. The lack of these processes significantly reduces the ability of social media to activate we-mode cognition that can expand each individual's potential for social understanding and action.^58^ Emotional sharing is affected, too (see Table 6 for further details),^59^ through emotional contagion (causing positive and negative feelings) and social comparison (causing negative feelings).

**Table 6.** Emotional Contagion and Social Comparison in Social Media

On one side, in social media, emotional states can be transmitted to others through emotional contagion,^106^ causing people to experience the same emotions without their knowledge, as demonstrated by a large-scale research involving 689,003 users on Facebook. Social comparison, on the other hand, causes envy and a sense of having wasted time, both of which are linked to a lower degree of well-being.^59^
Moreover, social media provide social rewards, through reputation enhancement (i.e., “likes”) and social connections (i.e., a friend request), increasing their hedonic and utilitarian value.^107^ In other words, social media provide a “QSE”^108^ that affects both behavior and neural responses. An fMRI study^108^ demonstrated that observing photos with many (compared with few) likes in social media was associated with greater activity in neural regions—the precuneus, medial prefrontal cortex, and hippocampus, as well as the inferior frontal gyrus—implicated in reward processing, social cognition, imitation, and attention. These results suggest that the quantification of reputation enhancements offered by social media influences how users perceive and respond to their content. In other words, they act as a tool for achieving/expressing peer influence, guiding individuals in the discovery of their social environment.^109^

QSE, Quantifiable Social Endorsement.

Finally, social media, for their technologically mediated nature, have crucial elements of the environment, which are not only beyond agential control but are also oriented to creating a platform that is addictive and is aimed at marketing products to its users.^59^

The final outcome of these processes is that social motivation—the psychological dispositions and biological mechanisms that condition the individual to preferentially orient to the social world, take pleasure in social interactions, and foster and maintain social bonds^60^—is significantly reduced in social media.

Nevertheless, as noted by different researchers,^8,59,61^ individuals are together online and feelings of participation, interaction, and togetherness emerges during the use of social media. How?

Unlike physical communities that permitted interaction between people from varied origins and stories, digital communities are always made up of people who have the same interests and knowledge. As suggested originally by Dohény-Farina,^9^ communities in social media are communities of practice, based on common interests. In fact, these communities share three fundamental characteristics^62^: a mutual commitment, a common enterprise, and a shared repertoire of interpretive resources. These three factors generate a “common ground,” a set of shared beliefs, emotions, goals, and knowledge,^63^ which is the result of implicit, narrative, and situated learning to be and act as a member of the community. Finally, this common background is continuously updated by the community using a process of explicit sharing of knowledge and their related emotions (“grounding”) referred to the common goals of the community.

Unfortunately, social media significantly alter the process of grounding (see Table 7 for further details), disrupting the “wisdom of crowd” of the communities of practice^64^: the digital community becomes more radical and less accurate (polarization effect), allowing influential users to have disproportionate influence over the community's beliefs (social capital distribution).

**Table 7.** The Process of Grounding in Social Media

In social media, two factors significantly influence the process of grounding^21,110^: fearfulness and reputation enhancements.
As we have seen previously,^21^ fearfulness is a key adaptive affective trait supporting the levels of cooperative concern and care. And given the power of social media in generating emotional contagion, collective emotions of social media communities play a critical role in driving their decision. Chung and Zheng^110^ demonstrated that in these communities, influential users tend to express intense emotions of fear, anger, disgust, and sadness. In particular, fearful emotions (anger and fear) are very effective in driving influence in social media communities.
However, given the influence of reputation enhancements, communities of practice in social media tend to be centralized: a small number of people at the “center” of the community are connected to many more individuals in the “periphery.” So, more frequently from what happens in physical communities, ideas are filtered through a powerful social influencer.
Finally, Cheng et al.^80^ pointed out that the characteristics of digital communities generate an unequal distribution of social capital. In them, social anxiety is caused by being overly sensitive to flaws in one's public behavior, which might make other people criticize them, while loneliness is caused by the feeling that one's interpersonal relationships are not as they would like them to be. Putting them together, social enhancement is linked to high preferences for, but low problems, in social relationships, while social compensation is linked to high problems in social relationships and high preferences for online social relationships. Summarizing, levels of online social capital go up when there are no relationship problems (like high social competence and low social avoidance) and when people use social media actively (e.g., psychological engagement, self-expression). On the other hand, these benefits do not happen when there are problems in relationships (like high social deficits or a lot of conflict between people), when people use social media passively, or when both things happen.

At the same time, although the lack of physical contact between their members generates emotional contagion, but not empathy, this has a negative impact on the development of extended affectivity, which, as we have seen, is a key component of the “we-mode.” On the one hand, not having empathy makes it harder for a person to interact directly with others in the community. This makes it easier for a person to experience the community in a passive way, such as monitoring other people's lives without direct engagement. On the other hand, the members of digital-only communities do not experience the main effect of empathy in physical communities^65^: distress reduction. Distress reduction happens when an observer feels the same emotions as the actor, and the observer helps the actor to ease its own distress, The final result is paradoxical^66,67^: many members, but not all, of digital communities feel alone together.

### Metaverse: digital communities meet the “we-mode”

In 2021, Mark Zuckerberg proposed a new era for the Internet, in which individuals would be immersed in a new digital experience known as the metaverse.^68–70^ In his words, the “deep sense of presence” that comes from the fusion between the virtual world and the physical one should transform our communication and social connections. How? The experience of presence is enabled by one of the metaverse's less evident features: the metaverse operates similar to our minds.^12,69^

Cognitive sciences have long regarded the brain as a computer capable of processing and describing information. Although this viewpoint continues to impact popular thinking, neuroscience now compares our brain to a simulator, a mental virtual reality (VR) system that has evolved to anticipate sensory events before they are perceived (predictive coding).^71,72^

The same is true for the metaverse.^73^ In fact, both VR and augmented reality (AR)—the key technologies of the metaverse—try to predict how the user's actions will affect their senses by creating the same scene (seen through the helmet) and feelings (generated by sensors) that the user would feel in the real world. In this view, the sense of presence comes from the metaverse's ability to predict how the mind simulates reality and to make digital content that matches these predictions.^74^

The more accurate the prediction, the more the person in the virtual environment will feel like they are really there, even though they know it is not real.^75^ Different studies (see Table 8 for more information) have shown that VR and AR can trigger most of the “we-mode” neurological processes that happen in physical communities.^76^ If the many flaws in this technology are fixed,^77,78^ it might encourage people to engage in more meaningful and constructive interactions in online communities.

**Table 8.** “We-Mode” in the Metaverse

As demonstrated recently, the interaction in the metaverse produces a strong sense of presence and social presence (“we-mode”) by activating some of the same neuroscience processes—activation of place cells, empathy, oxytocin production, and interbrain synchrony—typical of physical communities.
First, different studies on rats^111,112^ demonstrated that VR is able to generate place-cell activation. Specifically, hippocampus place-cell activity with similar features to those recorded in real environments was elicited throughout the VR experience.
Second, Martingano et al.^113^ investigated the effect of VR experiences on users' empathy in a recent meta-analysis that included 43 different studies. Their findings revealed that seeing evocative stimuli in VR can promote emotional empathy in a variety of ways.
Moreover, in a recent study, Dekker et al.^114^ explored the difference between VR and traditional 2D pornography movies in generating intimacy and illusion of interaction with the porn actors. Intriguingly, saliva oxytocin levels were correlated with the sense of eye contact with virtual people, suggesting a role for the social neuropeptide in the perception of heightened intimacy and connection in VR.
Finally, different studies^115,116^ from the research group directed by Mark Billinghurst proved that interbrain synchrony in virtual environments may be achieved in a manner comparable to that seen in the actual world. Eye gaze and visual perspective, in particular, play an important role in eliciting and enhancing interbrain synchrony in VR.

2D, two dimensional; VR, virtual reality.

## Conclusions

According to Tuomela,^10^ what defines a community is the “we-mode”: working together as a “we” to promote the interests of “us.” In we-mode, individuals must intend to behave or have attitudes as a group for the same group purpose (collective intentions), and they must see themselves as members of a community.

For a long time, communities developed only within places. Places, through their boundaries, provide a physical border to the activity of members of the community. Moreover, as recently demonstrated by neuroscience, they also activate different we-mode neurobiological and cognitive processes—behavioral synchrony, joint attention, intentional attunement, interbrain synchronization, and so on—that alter the representation of the interactive scene, providing a broader understanding of the behavior of the involved individuals, and thus of their available options for action.^79^

Cooperative and collective behaviors have two further effects. First, through them, the members of the community develop an interbrain synchrony that further reduces their psychological distance. Second, they provide the basis for the development of shared narratives that transform the autobiographical memories of collective actions into collective memories, bridging the individual with the community. Specifically, collective memories generate in the individual the feeling of being a member of the community, developing a sense of social identity.

Taken together, all these neurobiological and cognitive processes also have a significant effect on the generation of social capital, in particular on the bonding one. By reducing the psychological distance between the members of the community, through the activation of the we-mode, they generate a feeling of social cohesion that unites them. In summary, physical communities generate cohesive social networks composed of individuals who can be very different from each other.

Digital communities, instead, are significantly different. Digital technologies, by removing the physical boundaries that define a place, allow greater freedom in the behavior of individuals and the selection of community members, maximizing the bridging social capital. At the same time, however, the lack of physical co-presence of community members significantly reduces their possibility of activating we-mode cognitive processes and their overall level of social motivation. For this reason, digital communities are communities of like-minded individuals, based on common interests and shared knowledge (communities of practice).

Given the peculiar characteristics of social media, digital communities of practice are significantly different from physical ones, too. In particular, the process of grounding is influenced by two factors—fearfulness and reputation enhancements—that can disrupt the “wisdom of crowd,” reduce the well-being of their members, and allow influential users to exert disproportionate influence over the beliefs of the community. Finally, these characteristics also generate an unequal distribution of social capital.^80^

In conclusion, the emergence of digital communication and social media is transforming the structure and experience of communities. Communities are born less and less in places and more and more in social media platforms. This makes it harder for people to experience “we-mode” cognitive processes and their significant advantages, which are a key part of our evolutive tools.

A new emerging technology—the Metaverse^69^—may reverse this course by allowing the activation of the “we-mode” also in digital communities. However, the metaverse's beneficial applications are only one side of the coin. The risks^77^—privacy, security, fraud, dependence, and so on—implied by this technology must be addressed before it can be fully deployed.
